# Intravenous lidocaine to prevent endothelial dysfunction after major abdominal surgery: a randomized controlled pilot trial

**DOI:** 10.1186/s12871-020-01075-x

**Published:** 2020-06-23

**Authors:** Marco Pustetto, Nicolas Goldsztejn, Karim Touihri, Edgard Engelman, Brigitte Ickx, Luc Van Obbergh

**Affiliations:** 1 Department of Anesthesiology, Centre Hospitalier Universitaire Grenoble-Alpes, Boulevard de la Chantourne, 38700 Grenoble, France; 2 Department of Anesthesiology, Erasme Hospital, Université Libre de Bruxelles, Brussels, Belgium; 3 Department of Anesthesiology, CHIREC Hospital group, Brussels, Belgium

**Keywords:** Endothelial glycocalyx, Lidocaine, Major abdominal surgery, Endothelial dysfunction

## Abstract

**Background:**

Major abdominal surgery is associated with endothelial glycocalyx disruption. The anti-inflammatory effects of lidocaine were recently associated with endothelial barrier protection.

**Methods:**

This was a single-centre, parallel group, randomized, controlled, double blind, pilot trial. Forty adult patients scheduled for major abdominal surgery were included between December 2016 and March 2017 in the setting of a University Hospital in Brussels (Belgium); reasons for non-inclusion were planned liver resection and conditions associated to increased risk of local anesthetics systemic toxicity. Patients were randomized to receive either lidocaine by continuous intravenous administration or an equivalent volume of 0.9% saline.

The primary endpoint was the postoperative syndecan-1 concentration (difference between groups). Near-infrared spectroscopy of the thenar eminence in association with the vascular occlusion test, and contemporary analysis of flow-mediated dilation of the brachial artery were the secondary outcomes, along with haemodynamic data. Blood samples and data were collected before surgery (T0), and at 1–3 h (T1) and 24 h (T2) post-surgery.

**Results:**

Syndecan-1 concentration increased significantly post-surgery (*P* < 0.001), but without any difference between groups. The near-infrared spectroscopy-derived and flow-mediated dilation-derived variables showed minor changes unrelated to group assignment. Compared with the placebo group, the intervention group had a significantly lower peri-operative mean arterial pressure and cardiac index, despite equally conducted goal-directed haemodynamic management. Postoperative lactate concentrations were similar between groups.

**Conclusions:**

Lidocaine failed to have any effect on endothelial function. Since in comparisons to other types of clinical situations, syndecan-1 was only slightly upregulated, endothelial dysfunction after major abdominal surgery might be overestimated.

**Trial registration:**

« *ISRCTN Registry »* identifier: ISRCTN63417725. Date: 15/06/2020. Retrospectively registered.

## Background

Amide-linked local anesthetics (LAs) such as lidocaine and ropivacaine have an anti-inflammatory effect [1–5]. Their use in the peri-operative setting for this purpose was proposed more than one decade ago [1–5]. Continuous intravenous infusion of lidocaine reduced pain scores after major abdominal surgery, improves gastrointestinal recovery and potentially reduces the in-hospital length of stay [6]. Whether these effects are related to sodium-channel blockade is debated. However, the inhibition of tumor necrosis factor alpha (TNF-α) signaling pathway by LAs was recently associated with endothelial barrier protection in vitro [5].

The endothelial glycocalyx (EG) is a key component of the endothelial surface layer. It is essential for the regulation of the vascular barrier function, interaction between endothelial cells and blood cells, and in the transmission of shear stress. Extensive tissue trauma, hypervolemia, systemic and regional ischemia–reperfusion injury and shock from any origin have been associated with EG disruption. Systemic inflammation appears to have a causative role in EG shedding, with the exception of acute hypervolemia, for which the primary insult may be a direct mechanical injury [7, 8]. However, a direct cause-effect relationship between inflammation and EG shedding is difficult to establish. Damage of the EG leads to tissue edema (i.e. decreased tissue access to oxygen and nutrients), increased interaction with leukocytes and platelets, and increased inflammation in a vicious cycle [7].

The same clinical scenarios are often associated with notable microcirculatory alterations. In these settings, markers of EG disruption and microvascular derangement are correlated with patients’ morbidity and mortality [9–11]. Major abdominal surgery represents a scheduled but severe tissue trauma, is associated with important fluid shifts and an inflammatory state and actually induces EG shedding and disturbs endothelial function [12, 13].

Little information exists regarding possible restoration of the EG. To date, the most effective therapeutic strategy may be to preserve endothelial function and the EG; many different interventions have been proposed [10, 14–17]. Some of these are reasonable fluid administration [14], use of anti-inflammatory drugs [15], and possibly goal-directed haemodynamic management [18]. The results achieved with hydrocortisone [15] support the idea that inflammation could be at least one mechanism that disrupts the EG. Anti-inflammatory drugs may therefore be used for this specific purpose. We hereby hypothesise that intravenous continuous infusion of lidocaine can protect the EG and preserve endothelial function during major abdominal surgery.

## Methods

### Trial design

This was a randomized controlled pilot trial that compared two parallel groups for superiority of intervention versus placebo. Ethical approval for this study (Ethics Committee No. P2016/404/2016–003918-27) was provided by the Ethics Committee Erasme Hospital, 808 route de Lennik, B-1070 Brussels, Belgium (Chairperson Prof J.-M. Boeynaems) on 24 October 2016. The study was retrospectively registered in the ISRCTN Registry (Identifier: ISRCTN63417725) the 15/06/2020. All included patients signed a written informed consent before participation. This study adheres to CONSORT guidelines for reporting clinical trials (see Additional file 4: « CONSORT Checklist »).

### Participants

Adult (> 18 years old) patients scheduled for elective major abdominal surgery were investigated for eligibility. Patients scheduled for colonic or bariatric surgery were considered to have a ‘moderate risk’, and thus not included in the eligibility screening. Patients scheduled for hepatic resection were excluded to prevent potential accumulation of lidocaine because of unpredictable changes in its pharmacokinetics. Patients who were to be managed with combined epidural and general anesthesia (e.g. esophagectomy) were excluded because of potential parallel administration of another LA. Patients presenting one or more of the following medical conditions were excluded because of increased risk of lidocaine accumulation and/or local anesthetics systemic toxicity: severe heart conduction blocks without implantable pacemaker, severe liver and kidney insufficiency (Kidney Disease: Improving Global Outcomes [KDIGO] stage >3a), acute heart failure, and known allergic reactions to any amide-linked LAs. Patients with atrial fibrillation were also excluded because it was impossible to follow the fluid administration protocol (discussed in the ‘Interventions’ section).

### Study setting

This study was conducted at Erasme Hospital (Brussels, Belgium), a tertiary healthcare institution of the Université Libre de Bruxelles. Patients were enrolled from December 2016 to March 2017.

### Interventions

**Fig. 1:**
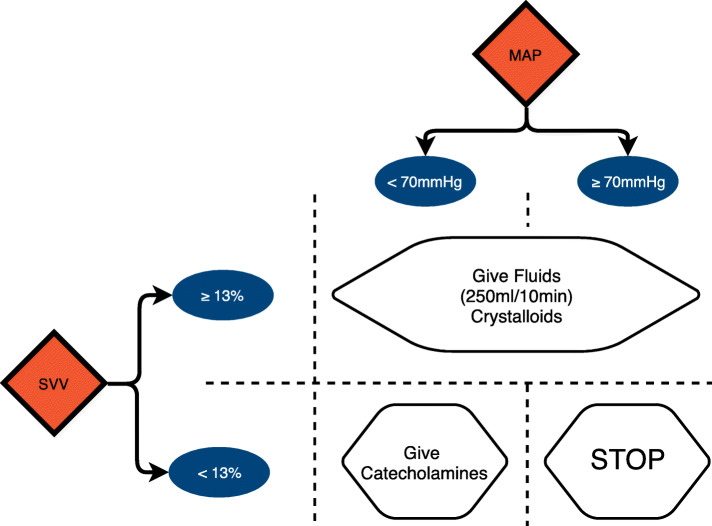
Goal-directed haemodynamic protocol. Goal-directed protocol for the management of peri-operative fluids and haemodynamics: systolic volume variation (SVV) and mean arterial pressure (MAP), as indicated on FloTrac™ (Edwards Lifesciences Corp., Irvine, CA, USA)

Patients allocated to the intervention (LIDO) group received 1.5 mg kg^− 1^ (total body weight [TBW]) of 1% lidocaine (Xylocaïne®; AstraZeneca, Cambridge, United Kingdom) just before anesthesia induction, which was immediately followed by a 2 mg kg^− 1^ h^− 1^ (TBW) continuous intravenous infusion until skin closure. Patients allocated to the placebo (PLA) group received an equivalent volume of 0.9% saline (0.15 ml kg^− 1^ bolus and 0.2 ml kg^− 1^ h^− 1^ continuous intravenous infusion). Anesthesia protocol was standardized for both groups. When indicated, an intrathecal injection of 0.1–0.3 mg of morphine was administered before induction. The latter was achieved using propofol, remifentanil (with target-controlled infusion [TCI]) and a neuromuscular blocking agent (usually rocuronium or cisatracurium). Every patient received dexamethasone (10 mg). Maintenance was achieved using desflurane and remifentanil (TCI), guided to maintain the Bispectral Index (BIS™; Aspect Medical Systems, Norwood, MA, USA) readings between 40 and 60 with 0% of burst suppression rate. Hemodynamics were managed with a goal-directed therapy (GDT) protocol, based on stroke volume variation (SVV, measured using the FloTrac™ system [EV1000; Edwards Lifesciences Corp., Irvine, CA, USA]) and mean arterial pressure (MAP). Plasmalyte® (Baxter, Deerfield, IL) administered at 2 ml kg^− 1^ h^− 1^ was the basal infusion. Triggers for fluid bolus administration (250 ml of crystalloids in 10 min) and for catecholamine optimization were SVV ≥ 13% and MAP< 70 mmHg (Fig. 1), respectively. When to transfuse hemoderivates and administer colloid solutions and the management of postoperative analgesia were at the discretion of the anesthetist in charge of the patient.

### Data collection

Endothelial function and the EG were investigated using three techniques: concentration of syndecan-1, measurement of the tissue oxygen saturation (StO_2_) during the vascular occlusion test (VOT), and contemporary measurement of flow-mediated dilation (FMD). Data and blood samples were collected before surgery (T0), at 1–3 h post-surgery in the recovery room (T1) and 24 h post-surgery in the surgical ward (T2).

The concentration of syndecan-1, a marker of EG shedding, was measured by classic sandwich enzyme-linked immunosorbent assay (ELISA), based on the manufacturer’s instructions (Syndecan-1 ELISA kit; Tebu-bio, Boechout, Belgium). Blood samples were collected in dry tubes, centrifuged to obtain the serum and stored at − 80 °C for a maximum of 6 months before final analysis.

The StO_2_ was measured continuously (every 2 s) and noninvasively using near-infrared spectroscopy (NIRS) (ForeSight®; CASMED®; CAS Medical Systems, Inc., Branford, CT, USA) on the thenar eminence, as previously described [11]. At the same time, the diameter of the brachial artery and flow velocity were measured continuously by using a 5-12 MHz linear ultrasonography transducer (Sparq®; Phillips, Amsterdam, the Netherlands), which was applied to the upper arm with mechanical support for image stabilisation (Image 1 in « Additional File 1 » shows the standard set up for a participant). Baseline values were acquired over 1 min. A VOT was then conducted by rapidly inflating a pneumatic cuff, which was placed around the forearm, up to 200 mmHg (or 50 mmHg suprasystolic pressure). After 5 min the cuff was released, and the hyperemic response was evaluated for another 4 min. The following StO_2_-derived variables were analysed: StO_2_-baseline, StO_2_-ischemic slope and StO_2_-reperfusion slope (Image 2 in « Additional File 2 » shows the evolution of StO_2_ during the test in one participant). The FMD was assessed by automated edge detection software (FMD Studio™ (CardioVascular Suite™); Quipu srl, Pisa, Italy) [19], based on the experts’ guidelines [20].

The following FMD-derived variables were analysed: brachial artery baseline diameter, FMD-maximum (i.e. the maximal diameter during reperfusion) and the area under the curve of estimated shear rate of hyperaemic flow until FMD-max (Image 3 in « Additional File 3 » shows the evolution of the brachial artery diameter and shear rate during a test in one participant).

Data concerning fluid requirements were prospectively collected during surgery and during recovery in the postanesthesia care unit (PACU). Haemodynamic variables were collected only during surgery by the EV1000 clinical platform.

### Outcomes

The primary endpoint was the evolution of the syndecan-1 concentration postoperatively in the LIDO group, compared with the PLA group (hereafter referred to as ‘difference between groups’). Predefined secondary outcomes were the effect of lidocaine on NIRS- and FMD-derived variables (i.e. difference between groups); the influence of surgery on NIRS and FMD-derived variables and its association with group assignment (hereafter referred to as ‘difference between times’); the correlation between glycocalyx, microcirculation and vascular reactivity at three time points; and the influence of group assignment on fluid requirements. Potential harmful effects of lidocaine were also systematically researched and reported. Haemodynamic variables, even if not originally included in secondary endpoints, were also taken in account for analysis and validation of compliance to the GDT protocol.

### Sample size

We were unable to find any published study on the effects of lidocaine on endothelial function in the clinical setting, and evidence concerning alteration of EG in major abdominal surgery is scarce. Hence, we decided to perform a pilot study which included 40 patients with 20 patients for each group.

### Randomisation

Participants were randomly assigned to one of two groups in a 1:1 ratio, based on Efron’s biased coin randomisation procedure generated with NCSS v10 Statistical Software (2015, NCSS, Llc. Kaysville, UT, USA).

### Blinding

Patients, healthcare providers, data collectors and outcome adjudicators were all blinded to group assignment. The physician in charge for generation of allocation sequence and concealment was not directly implicated in treatment administration or data collection.

### Statistical analysis

Data are presented as the mean ± the standard deviation. Data were compared between the groups using the Mann–Whitney test or by two-way analysis of variance (ANOVA) for repeated measures, as indicated. One factor was the study group and a second factor was time. For each variable, the three null hypotheses of the two-way ANOVA tests were that the means of the observations grouped by one factor would be the same; that the means of the observations grouped by the other factor would be the same; and that there would be no interaction between the two factors. The *P* value would be indicated for the difference between groups (i.e., all time points together) or for the difference between times (i.e., all groups together), and for the interaction between groups and times. A Tukey-Kramer multi comparison test was performed to examine all pairs of treatment means. The other continuous non longitudinal variables were compared with the Mann-Whitney test. For all tests, *P* < 0.05 was statistically significant. These computations were performed using the software package Systat version 5.0 for DOS (Systat, Inc., Evanston, IL, USA).

## Results

**Fig. 2:**
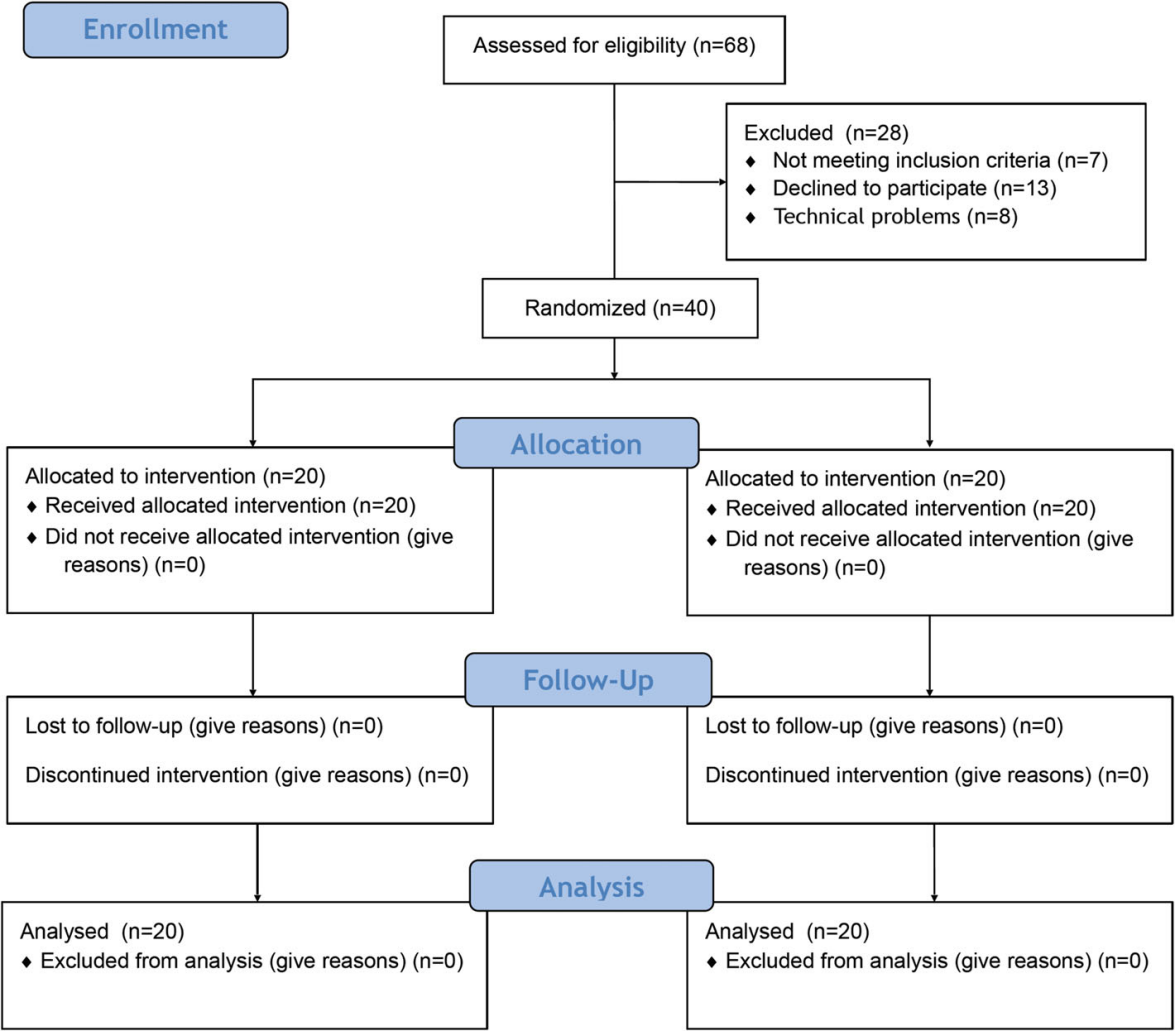
CONSORT flow diagram. CONsolidated Standards of Reporting Trials (CONSORT) flow diagram showing patients’ recruitment and allocation

We assessed 68 patients for eligibility and excluded 28 patients (seven patients had ≥1 exclusion criteria, 13 patients refused to participate, and eight patients were excluded for technical problems related to the connection between the ultrasonography transducer and the FMD detection software and thus the impossibility to perform measurements on the brachial artery). Trial recruitment was stopped when 40 patients were included, 20 for each group, as planned. All patients were included in the final analysis (Fig. 2).

The patients’ baseline characteristics for both groups are summarised in Table 1. Comorbidities and medications that influence baseline endothelial function and FMD measurements are reported in Table 1. The type of surgical intervention and whether it was conducted by laparotomy, laparoscopy or a combined procedure are reported in Table 2. Primary and secondary outcomes are presented in Table 3. Analysis of fluid requirements and hemodynamics are reported separately in Tables 4 and 5.

**Table 1:** Population characteristics. Placebo (PLA) group and lidocaine (LIDO) group, data as mean ± SD or n (%)

	PLA (*n* = 20)	LIDO (*n* = 20)
Age *(years)*	60 ± 10.9	63 ± 12.5
Male gender	10 (50)	13 (65)
Weight *(kg)*	74 ± 16.5	77 ± 14.2
BMI *(kg/m2)*	26 ± 5.6	26 ± 4.9
ASA 1	1 (5)	1 (5)
ASA 2	15 (75)	12 (60)
ASA 3	4 (20)	7 (35)
POSSUM (physiologic)	16.9 ± 3.8	19.7 ± 4.1
POSSUM (operative)	12.4 ± 4.5	11.4 ± 2.9
POSSUM predicted morbidity (%)	32.4 ± 23.2	37.2 ± 18.6
POSSUM predicted mortality (%)	6.4 ± 9.8	6.8 ± 6.0
Cardiac Risk (LEE criteria) (%)	1.9 ± 2.4	1.4 ± 1.8
**Comorbidities**		
Arterial Hypertension	9 (45)	9 (45)
Hypercholesterolemia	4 (20)	3 (15)
Diabetis (typeI or II)	3 (15)	5 (25)
Ischemic cardiopathy	3 (15)	2 (10)
Other cardiopathy	0	2 (10)
Tobacco abuse	6 (30)	6 (30)
Renal Insufficiency (KDIGO stage <3b)	1 (5)	3 (15)
COPD	3 (15)	3 (15)
Stroke or TIA	1 (5)	2 (10)
Peripheral Arterial Disease	1 (5)	0
**Medications**		
Beta-blockers	2 (10)	7 (35)
Ca-channel blockers	2 (10)	0
ACE - inhibitors	7 (35)	4 (20)
Nitrates	0	0
Oral Antidiabetic Agents	1 (5)	3 (15)
Insuline	2 (10)	2 (10)
Other Medications	17 (85)	11 (55)

**Table 2:** Surgery performed. Placebo (PLA) group and lidocaine (LIDO) group, data as mean ± SD or n (%)

	PLA (*n* = 20)	LIDO (*n* = 20)
Laparotomy	11 (55)	12 (60)
Laparoscopy	4 (20)	5 (25)
Both	5 (25)	3 (15)
**Interventions**		
Total Mesorectal Excision	3 (15)	2 (10)
Gastrectomy (total or partial)	2 (10)	3 (15)
Duodeno-Cephalo-Pancreatectomy	8 (40)	4 (20)
Abdominoperineal resection	1 (5)	1 (5)
Surrenalectomy	1 (5)	1 (5)
Ovarian cancer cytoreductive surgery	2 (10)	1 (5)
Extensive retroperitoneal lymphadenectomy	0	1 (5)
Nephrectomy (total or partial)	2 (10)	6 (30)
Cystectomy	1 (5)	1 (5)
Duration, skin-to-skin (min)	232 ± 89	228 ± 80

**Table 3:** Main results. Endothelial Glycocalyx (EG), NIRS-derived (NIRS) and FMD-derived (FMD) variables, before surgery (T0), 1–3 h after arrival in PACU (T1) and 24 h after surgery (T2); data as Mean ± SD; analysis by 2-way ANOVA for repeated measures and Tukey-Kramer multi comparison test

		PLACEBO GROUP			LIDOCAINE GROUP			*P* value for difference between groups	*P* value for difference between times	*P* value for interaction
		T0	T1	T2	T0	T1	T2			
**EG**	Syndecan (pg/ml)	2896 ± 2558	3452 ± 2656***	3999 ± 3453**$	2115 ± 2007	2770 ± 2898**	3169 ± 2906*** $	0.179	<0.001	0.454
**NIRS**	StO_2_ - Baseline (%)	69.2 ± 3.1	70.7 ± 7.5	66.4 ± 5.1**	68.1 ± 5.8	70.1 ± 4.9	68.0 ± 5.0**	0.939	0.046	0.374
	StO_2_ - Ischemic slope (%/sec)	−0.165 ± 0.049	−0.147 ± 0.063*	−0.141 ± 0.041	−0.163 ± 0.042	− 0.145 ± 0.050*	−0.156 ± 0.038	0.551	0.021	0.464
	StO_2_ - Reperfusion slope (%/sec)	1.990 ± 0.449	1.672 ± 0.585	1.843 ± 0.525	2.030 ± 0.318	1.868 ± 0.397	1.986 ± 0.478	0.189	0.390	0.579
**FMD**	Baseline vascular diameter before occlusion (mm)	4.049 ± 0.684	4.367 ± 0.692	4.276 ± 0.605	4.223 ± 0.808	4.428 ± 0.744	4.610 ± 0.719	0.528	0.002	0.803
	Post-ischaemic diameter increase: FMD_max_ (%)	6.1 ± 3.9	3.6 ± 3.7	3.8 ± 4.7	6.9 ± 5.8	4.9 ± 6.7	6.6 ± 6.7	0.240	0.382	0.123
	AUC_ShearRate_ until FMD_max_ (arbitrary units)	21,512.6 ± 11,143.0	21,140.7 ± 13,084.5	16,527.8 ± 8734.7	16,270.9 ± 8397.2	18,588.7 ± 11,874.1	16,473.5 ± 8865.4	0.719	0.226	0.377

Inside each group: *** T0 vs T1 and T0 vs T2 *p* < 0.001; ** T1 vs T2 *p* < 0.02; * T0 vs T1 *p* < 0.05; ^$^ T1 vs T2 *p* < 0.01

**Table 4:** Fluid requirements and per-operative haemodymics. Fluid requirements (FLUIDS); peri-operative haemodymics (HD); data as Mean ± SD - Analysis by Mann-Whitney test

		PLACEBO GROUP	LIDOCAINE GROUP	*P* value for difference between groups
**FLUIDS**	Total in during surgery (ml)	3150 ± 1822	2410 ± 948	0.213
	Fluid balance during surgery (ml)	2237 ± 1408	1758 ± 957	0.267
	Total in during PACU stay (ml)	2820 ± 1208	2874 ± 1305	0.968
	Fluid balance during PACU stay (ml)	1613 ± 1200	1781 ± 1204	0.598
	Global fluid balance (ml)	3850 ± 1740	3540 ± 1469	0.607
**HD**	HR (bpm)	73.2 ± 11.6	68.7 ± 10.9	0.307
	MAP (mmHg)	78.8 ± 6.8	74.9 ± 4.1	0.033
	SVI (mL b^−1^ m^−2^)	39.9 ± 5.1	37.0 ± 8.8	0.088
	CI (L min^−1^ m^−2^)	2.9 ± 0.6	2.4 ± 0.4	0.024
	Percent of time passed with SVV < 13 (%)	66.1 ± 22.7	71.3 ± 26.0	0.335
	Percent of time passed with MAP ≥70 mmHg (%)	75.7 ± 19.0	70.8 ± 15.4	0.273
	Percent of time passed with CI ≥ 2.5 L min^−1^ m^−2^ (%)	68.8 ± 35.0	43.6 ± 32.9	0.025

**Table 5:** Point-of-care laboratory analysis. Point-of-care laboratory analysis (POCT); before surgery (T0); on arrival at PACU (T1); data as Mean ± SD; analysis by 2-way ANOVA for repeated measures

		T0	T1	T0	T1	*P* value for difference between groups	*P* value for difference between times	*P* value for interaction
**POCT**	Lactates (mmol/L)	0.91 ± 0.47	1.65 ± 0.97	1.04 ± 0.43	1.70 ± 0.92	0.646	< 0.001	0.734
	Glycemia (mg/dL)	122.4 ± 47.7	164.4 ± 44.2	126.8 ± 45.1	191.5 ± 50.6	0.237	< 0.001	0.113

We failed to show any difference between groups for primary and secondary outcomes. Syndecan-1 increased modestly and equally between groups, and maximum levels were registered at T2 (i.e. 24 h post-surgery). Difference between times was statistically significant (*P* < 0.001). There was no interaction between groups and times.

The StO_2_-ischemic slope decreased after surgery (*P* value for differences between times = 0.021; inside each group: T0 vs T1 = *P* < 0.02) without any influence of group assignment. The StO_2_-reperfusion slope was not significantly modified. Baseline brachial artery diameter before occlusion increased after surgery (*P* = 0.002), and was independent of group assignment. Maximal postocclusive dilation (i.e. FMD-max) tended to decrease at T1 and T2, without reaching statistical significance, because of high interindividual variability. Group assignment did not influence FMD-derived variables. Haemodynamic goals were achieved equally in both groups, and without any difference in cumulative fluid balance intraoperatively or until PACU discharge.

No serious adverse events occurred. Only two patients experienced a minor adverse effect (tinnitus) with lidocaine infusion, immediately after the loading dose. One patient developed a non-life threatening perioperative cardiac arrhythmia, but had been randomized to the PLA group. Mean arterial pressure (MAP) and cardiac index (CI) were significantly lower in the LIDO group; lactates on arrival at PACU were similar between groups (Tables 4 and 5). No other secondary effect occurred during intervention.

## Discussion

In this single-centre, pilot randomized controlled trial we tested the hypothesis that intravenous lidocaine could protect the EG and preserve endothelial function in 40 patients undergoing major abdominal surgery. Anesthesia and the patients’ management were strictly controlled and standardized between groups. Patients, healthcare providers and data collectors were blinded to group assignment. In this setting, lidocaine administration failed to show any effect on serum levels of syndecan-1 measured postoperatively or on NIRS- or FMD-derived variables.

Syndecan-1 concentrations increased at most 1.5-fold by 24 h post-surgery in a statistically significant manner (*P* < 0.001), and independently from group assignment. This moderate flaking of EG resembles the results described by Steppan et al. [12] in patients undergoing major abdominal surgery. However, it was much less pronounced than previously described in different clinical scenarios. Syndecan-1 was reported to increase up to 100-fold in trauma patients [21], 65-fold in patients undergoing major vascular surgery with global and regional ischemia [22], 8-fold in septic patients [12], and 3- to 4-fold after cardiac surgery [23, 24] or after resuscitated cardiac arrest [25].

Our results should be interpreted in the light of multiple considerations. First, even if the increase in serum levels of syndecan-1 were correlated with mortality [25], it is unknown whether this correlation would be true only beyond a given threshold. We consequently are unable to state if the 1.5-fold increase in syndecan-1 concentrations was clinically relevant. In addition, this focus was not an objective of the trial. Second, it is difficult to directly compare the results obtained from different populations (e.g. trauma, cardiac surgery, sepsis) and different mechanisms of primary insult. Moreover, even when a similar population is taken into account (e.g., major abdominal surgery), it should be emphasised that the study by Steppan et al. [12] lacked detailed information about surgical procedures and the patients’ baseline characteristics and perioperative management. Thus, making a direct comparison was not possible. Third, to our knowledge, this is the first randomized controlled trial that studied the effects of a drug on EG in a perioperative setting which had been optimized to reduce endothelial dysfunction. In fact, previous literature reports were primarily observational or focused on a single intervention without any attempt to control the plethora of factors that possibly influence EG disruption (in particular fluids, hemodynamics and corticoid administration) [8, 14, 17]. In our study, fluids and haemodynamic management were goal-directed and patients of both groups received 10 mg dexamethasone. The latter is the standard of care in our institution for postoperative nausea and vomiting prevention and is part of a multimodal strategy for pain control. It is possible that the pronounced and prolonged anti-inflammatory effect of dexamethasone could mask or attenuate any effect of lidocaine. This factor is actually a limitation of this study. However, we decided to maintain this strategy for ethical reasons and in the belief that clinical research should try to improve patients’ outcome beyond the best known clinical practice.

Near infrared spectroscopy may be used in combination with a VOT to assess the microcirculatory response to an ischemic challenge, and thus reflect the pre-existing vascular reserve [26]. This technique has been previously used in different clinical populations to assess peripheral microvascular adequacy. We were unable to show any influence of lidocaine on NIRS-derived variables. The StO_2_-baseline was slightly increased in the immediate postoperative period (T1) for both groups, possibly because of receiving oxygen therapy in the recovery room and an increased oxygen delivery to peripheral tissues. The StO_2_-ischemic slope was significantly different between times but not between groups. The StO_2_-ischemic slope reflects the balance between oxygen reserves and the metabolic rate of muscle leads under the NIRS sensor [27] (i.e. thenar eminence) and its decrease was possibly because of a decreased metabolic rate of immobile sedated patients in the immediate postoperative period [28]. The StO_2_-ischemic slope was correlated with StO_2_-reperfusion slope, owing to the influence of metabolite accumulation on local microvascular reactivity after ischemia [29, 30]; however, it seemed not to be the situation in this cohort. The StO_2_-reperfusion slope was comparable between groups and showed no relevant modification in the postoperative period. This finding is a strong argument against a substantial alteration of the microvascular reserve.

In the literature, the StO_2_-reperfusion slope was significantly slower in critically ill patients than in control subjects [31], in septic intensive care unit (ICU) patients than in non-septic ICU patients [32], or in patients undergoing cardiopulmonary bypass (CPB) [33], all conditions that are associated with a substantial microcirculatory impairment. Its decrease in these settings was correlated with a worst outcome [31–33].

With regard to microvascular reactivity, data on endothelial function tested using FMD did not support any effect of lidocaine infusion. The baseline diameter of the brachial artery was significantly increased in the postoperative period, probably because of anesthesia-induced vasodilation. The maximal postischemic dilation of the brachial artery (i.e. FMD-max) was similar in both groups. A trend of FMD-max decrease occurred in the early postoperative period, independently from group assignment. However, it did not reach statistical significance because of high intersubject variability. This result should be interpreted with caution because a significant increase in baseline diameter (approximately 7%) may result in a decrease in FMD that depends on changes in the resting tone rather than endothelial dysfunction [20]. Moreover, the baseline FMD results suggested that a proportion of patients may have had endothelial dysfunction before surgery, which could attenuate the effects of any intervention in the perioperative setting. Future studies may eventually address this topic further by dividing a population in subgroups, based on baseline analysis of endothelial function. A much more important decrease in FMD-max was recently reported after cardiopulmonary bypass with continuous flow (from 12.8 to 1.6%) [34], which is a setting associated with increased inflammation, EG disruption and endothelial dysfunction [23, 24]. Flow-mediated dilation may be intimately linked to EG ‘health status’, as demonstrated by Yen et al. [35] In their study, flow-induced endothelial nitric oxide production and thus vasodilation were markedly reduced after EG selective enzymatic disruption, which suggests that EG is a key component in transmitting shear stress to endothelial cells. Consistent with the fact that EG was only slightly degraded in our cohort, FMD-max was not expected to show major modifications. Moreover, the StO_2_-reperfusion slope was recently correlated with FMD-max in healthy volunteers [30]. It was not significantly altered in the study population. Syndecan-1, NIRS and FMD may be complementary means to investigate the same problem: global endothelial dysfunction. Even though we were unable to show a clear correlation between these parameters, to our knowledge this is the first randomized trial to investigate their relationship in the perioperative setting.

There was no significant difference in fluid balance between the two groups. Patients of both groups spent approximately 70% of surgery time inside the limits imposed by the GDT protocol. Anesthetist reactivity to FloTrac™ measurements, time necessary to administer fluids or catecholamines and time the patient needed to respond to the intervention probably accounted for 30% of time passed outside the protocol limits. Nevertheless, the LIDO group had a significantly lower overall MAP and CI than the placebo group. More patients in the LIDO group needed norepinephrine to maintain haemodynamic goals (85% in the LIDO group vs 60% in the PLA group), even though the total dose of norepinephrine in patients that actually received it was similar in both groups (data not shown). Consistent with the fact that CI was not a targeted haemodynamic variable, patients in the LIDO group spent only 43.6% of time with a CI ≥2.5 l min^− 1^ m^− 2^, compared with patients in the PLA group (68.8%). The variability of CI was higher than that of targeted variables (i.e. SVV and MAP). However, postoperative lactate increase on arrival in the PACU was similar in both groups. We did not record any other relevant secondary effect.

This study presents some limits. First, this was a small, single-centre, pilot trial. Our data may not be generalisable, particularly because of differences in surgical management. Second, the use of syndecan-1 as the primary outcome could be controversial because endothelial function is difficult to characterise in the clinical context and it is likely that a single measure is insufficient to describe its complexity. For this reason, we used both NIRS- and FMD-derived variables in an attempt to present a more comprehensive model for the clinical study of endothelial function. However, in our population we failed to demonstrate any major endothelial dysfunction, independent from group assignment and contrary to what was expected based on previous literature findings [8, 9]. Third, the lack of a positive control (i.e. patients with significant endothelial dysfunction and EG disruption) is a limit and prevented the ability to demonstrate any significant correlation between syndecan-1 concentrations, StO_2_-reperfusion slope and FMD-max, as initially hypothesised.

## Conclusions

Based on previous studies demonstrating the anti-inflammatory properties of amide-linked LAs [3] and its effects on bowel motility [2], we hypothesised that lidocaine could reduce fluid extravasation and tissue edema by protecting the EG. Even if this was a pilot study, with a reduced population number, endothelial dysfunction in major abdominal surgery is probably overestimated when studied in a controlled setting. Based on our results, the hypothesis that lidocaine could affect endothelial function is unlikely.

## Supplementary information

**Additional file 1.** Image 1 shows the standard set up for a participant.

**Additional file 2.** Image 2 shows the evolution of StO_2_ during the test in one participant.

**Additional file 3.** Image 3 shows the evolution of the brachial artery diameter and shear rate during a test in one participant.

**Additional file 4.** Consort checklist.

## Data Availability

The datasets generated and/or analysed during the current study are not publicly available due technical reasons, but are available from the corresponding author on reasonable request.

## Abbreviations

CI: Cardiac Index
EG: Endothelial Glycocalyx
FMD: Flow Mediated Dilation
GDT: Goal Directed Therapy
LAs: Locals Anesthetics
LIDO: LIDOcaine Group
MAP: Mean Arterial Pressure
NIRS: Near-Infrared Spectroscopy
PACU: Post-Anesthesia Care Unit
PLA: PLAcebo Group
StO_2_: Tissue Oxygen Saturation
SVV: Systolic Volume Variation
VOT: Vascular Occlusion Test
