# Supplementary figures and images for: Intravenous lidocaine to prevent endothelial dysfunction after major abdominal surgery: a randomized controlled pilot trial
Source: BMC Anesthesiol. 2020 Jun 23;20:155. doi: 10.1186/s12871-020-01075-x (PMC7310453; doi:10.1186/s12871-020-01075-x)

US: mode B + PW

US transducer

NIRS sensor

FMD analysis software

NIRS

blood pressure cuff

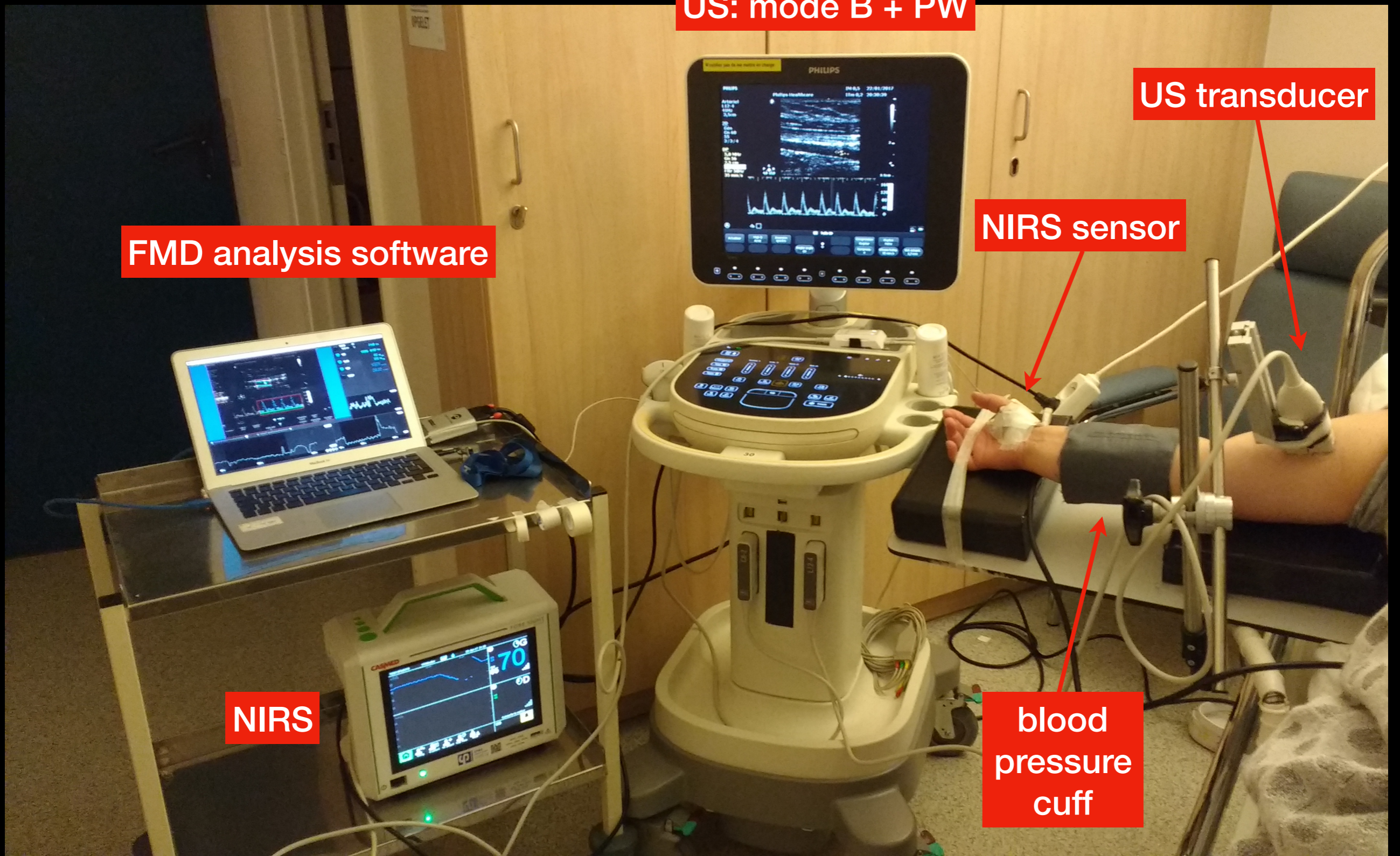

Supplement: Supplementary file 1 — Additional file 1. Image 1 shows the standard set up for a participant. [file 12871_2020_1075_MOESM1_ESM.pdf]

# NIRS - Patient 6/40

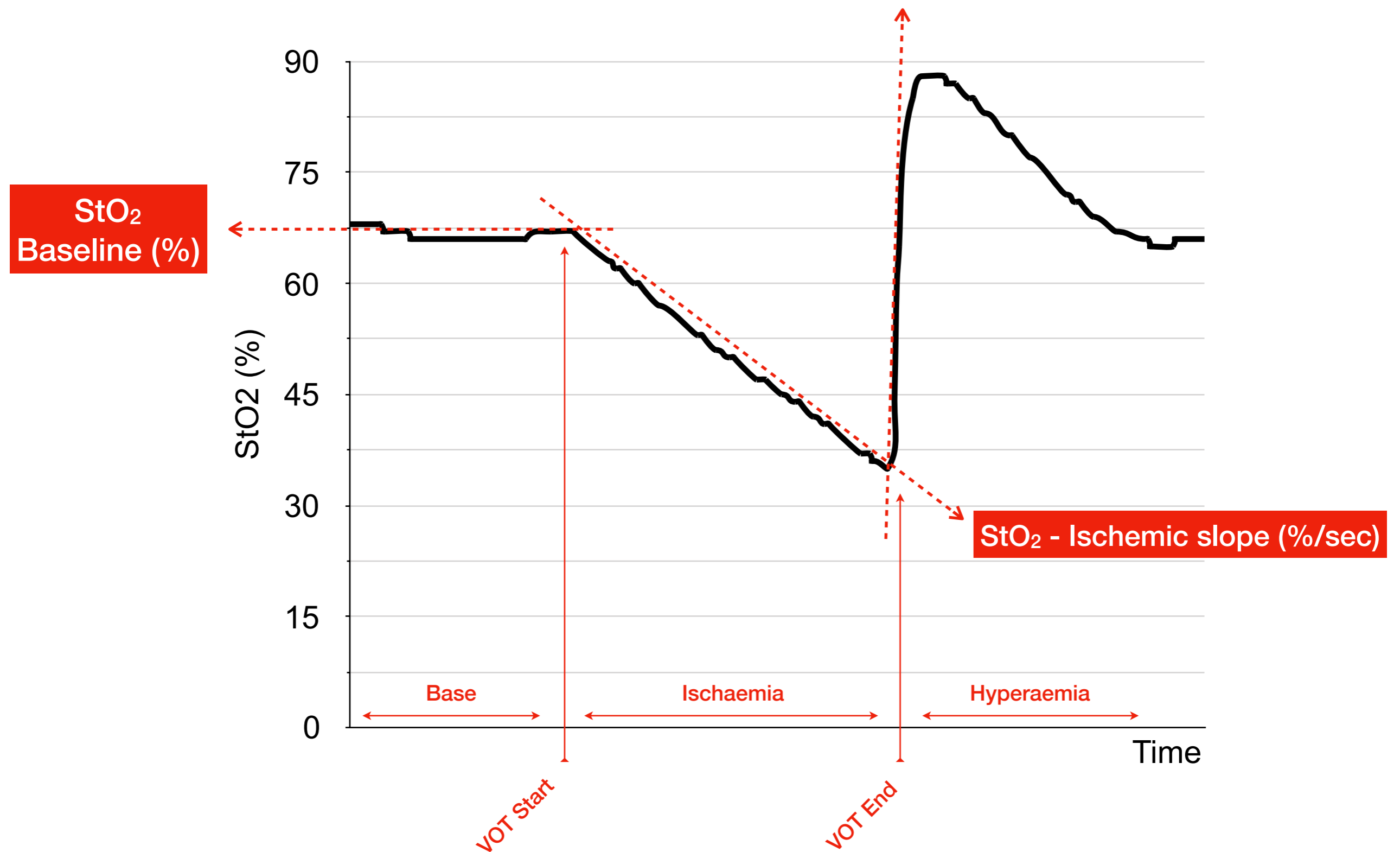

Supplement: Supplementary file 2 — Additional file 2. Image 2 shows the evolution of StO2 during the test in one participant. [file 12871_2020_1075_MOESM2_ESM.pdf]

# Diameter

Patient 6/40

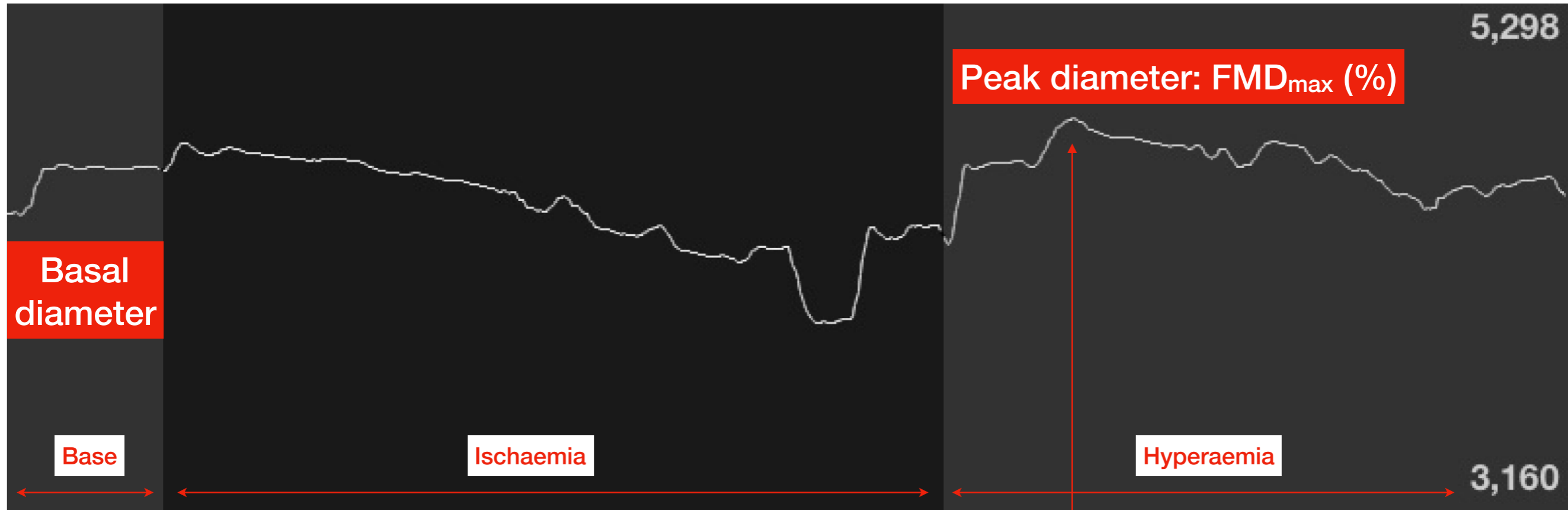

# Shear Rate

Patient 6/40

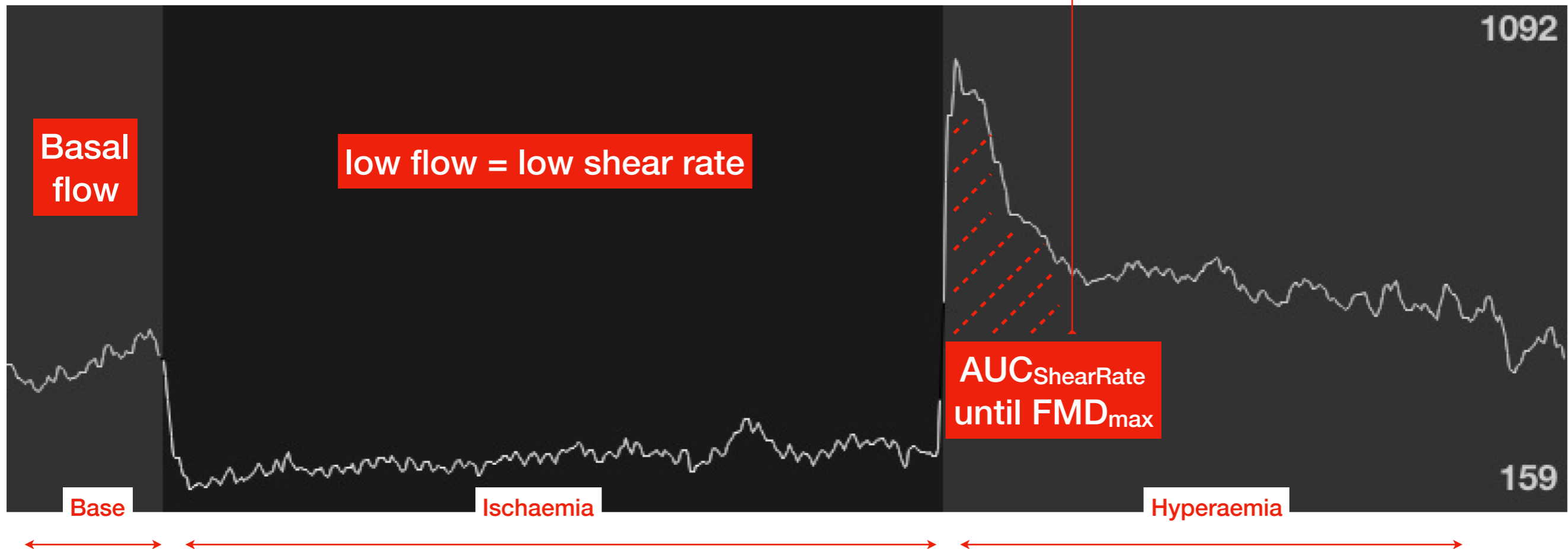

Supplement: Supplementary file 3 — Additional file 3. Image 3 shows the evolution of the brachial artery diameter and shear rate during a test in one participant. [file 12871_2020_1075_MOESM3_ESM.pdf]
